# Microvascular Changes in Exfoliation Syndrome: OCT Angiography Analysis of Peripapillary Density and Foveal Avascular Zone

**DOI:** 10.1155/joph/3508810

**Published:** 2026-04-03

**Authors:** Arzu Karakiraz, Şerife Bayraktar

**Affiliations:** ^1^ Department of Ophthalmology, Semdinli State Hospital, Hakkari, Turkey; ^2^ Department of Ophthalmology, Istanbul Faculty of Medicine, Istanbul University, Istanbul, Turkey, istanbul.edu.tr

**Keywords:** exfoliation syndrome, foveal avascular zone, glaucoma, pseudoexfoliation, vascular density

## Abstract

**Purpose:**

To investigate retinal vascular alterations in unilateral and bilateral pseudoexfoliation by assessing macular and peripapillary vascular density, as well as foveal avascular zone parameters.

**Patients and Methods:**

This prospective study included 77 eyes from 39 patients with unilateral pseudoexfoliation, 54 eyes from 27 patients with bilateral pseudoexfoliation, and 50 eyes from 25 healthy controls. All participants underwent OCT‐A imaging with the Topcon ImageNet 6 system. Only scans with signal quality ≥ 6/10 were analyzed. Superficial and deep retinal capillary plexus densities were quantified within a 6 × 6‐mm macular area, and foveal avascular zone dimensions were manually measured at the superficial plexus. Peripapillary vascular density was also evaluated. Statistical significance was set at *p* < 0.05.

**Results:**

Age and gender were comparable across groups (*p* > 0.05). Both unilateral and bilateral pseudoexfoliation eyes demonstrated significantly reduced macular superficial and deep capillary plexus densities in all quadrants except nasal (*p* < 0.05). Foveal avascular zone area and diameter were significantly enlarged in pseudoexfoliation groups compared with controls (*p* < 0.05). Peripapillary vascular density was decreased in all quadrants except nasal in both pseudoexfoliation groups (*p* < 0.05). No vascular differences were observed between unilateral and bilateral pseudoexfoliation eyes.

**Conclusion:**

Pseudoexfoliation is associated with widespread macular and peripapillary microvascular compromise, supporting its bilateral nature even in clinically asymmetric cases.

## 1. Introduction

Pseudoexfoliation syndrome (PXS) is an extracellular matrix disorder that affects both ocular and systemic organs. It is most commonly diagnosed through the identification of pseudoexfoliative material on the anterior lens capsule. This material typically accumulates in the lens capsule, ciliary body, corneal endothelium, zonules, iris, and trabecular meshwork [1]. While the precise pathophysiology of PXS remains incompletely understood, recent studies have advanced our knowledge of the underlying mechanisms. It is hypothesized that PXS represents an abnormal form of elastosis resulting from the excessive production of elastic microfibrils, particularly Fibrillin 1 [2].

The prevalence of PXS varies widely, ranging from 0.2% to 30%, depending on the population studied [3]. It typically presents as a bilateral and asymmetric condition. In addition to being a precursor to glaucoma, PXS is a significant risk factor for the progression of glaucomatous optic neuropathy. As such, patients with PXS require regular monitoring for glaucoma [4].

Optical coherence tomography angiography (OCT‐A) is a noninvasive technique that evaluates the microcirculation by assessing the movement of red blood cells. It is a highly reproducible, fast, and side‐effect–free diagnostic tool that can be used to assess peripapillary and macular vessel density [5].

The aim of this study is to investigate retinal and optic nerve vascular density changes, as well as to compare foveal avascular zone (FAZ) parameters, in patients with unilateral pseudoexfoliation material (PXF) detected clinically through slit‐lamp biomicroscopy, versus those with bilateral PXF. Furthermore, this study aims to evaluate whether patients with bilateral pseudoexfoliation show differences at the microvascular level and to assess subclinical microvascular changes in eyes without detectable PXF.

## 2. Patients and Methods

Patients diagnosed with PXS who presented to the general outpatient clinic and the glaucoma department of the Istanbul Faculty of Medicine, Department of Ophthalmology, between November 2023 and January 2024, were included in the study. Ethical approval for the study was subsequently obtained from the Istanbul Faculty of Medicine Ethics Committee. The study was initiated with ethical approval dated 11.12.2023, with approval number 2292648. Written informed consent was obtained from all participants prior to enrollment in the study.

A total of 131 eyes from 66 patients were included: 77 eyes from 39 patients with clinically diagnosed unilateral pseudoexfoliation, and 54 eyes from 27 patients with bilateral pseudoexfoliation. Additionally, 50 eyes from 25 age‐ and gender‐matched healthy volunteers were included as a control group. Eligible eyes from patients with unilateral and bilateral pseudoexfoliation were included in the analysis, and intereye correlation was accounted for using mixed‐effects models.

Inclusion criteria for the study were as follows: patients aged 40–85 years, with no history of systemic vascular or neurological diseases, and with PXF detected on the anterior lens surface and in the anterior chamber during slit‐lamp biomicroscopy. Subjects had no history of ocular surgery, a best‐corrected visual acuity (BCVA) of 0.5 or higher on the Snellen scale, and a spherical equivalent refractive error between +3 and −3 diopters. Patients with vitreous opacity, corneal opacity, or lens sclerosis that could reduce image quality were excluded. Only those with sufficient OCT‐A image quality to ensure proper analysis were included in the study.

Volunteers aged 40–85 years, with no history of systemic vascular or neurological diseases, normal biomicroscopic examination findings, no history of ocular surgery, and a BCVA of 0.5 or higher on the Snellen chart, with a spherical equivalent refractive error between +3 and −3 diopters, were included in the study to ensure optimal OCT‐A compatibility.

The BCVA of all participants was assessed using the Snellen chart, and their spherical equivalent refractive errors were calculated. A detailed biomicroscopic examination was performed on all participants, and intraocular pressures (IOPs) were measured using the Goldmann applanation tonometer. A detailed fundus examination was conducted using a 90D lens. Central macular thickness, subfoveal choroidal thickness, and retinal nerve fiber layer (RNFL) thickness were measured using SD‐OCT (Heidelberg Engineering, HRT + OCT Spectralis, Heidelberg, Germany).

Patients with glaucomatous damage identified during optic nerve examination and defects observed in RNFL measurements were further evaluated for secondary glaucoma due to pseudoexfoliation. These patients underwent a 24–2 automated visual field test (Humphrey Field Analyzer 2, Model 750, Zeiss, Oberkochen, Germany). Based on the Hodapp–Parish–Anderson criteria, patients were classified into mild, moderate, and severe stages of glaucoma. A mean deviation (MD) value greater than −6.0 was classified as mild‐stage glaucoma, an MD between −6.00 and −12.00 was classified as moderate‐stage glaucoma, and an MD less than −12.00 was classified as severe‐stage glaucoma.

All patients included in the study underwent optical disk and macula imaging using the OCT Topcon ImageNet 6 (DRI OCT Triton, Topcon Corporation, Japan). Patients with a signal quality of 6/10 or higher were included in the study. The optical disk and macula vessel density measurements were performed within a 6 × 6‐mm area. The FAZ area was manually calculated in square millimeters using the device’s own software for superficial retinal plexus measurements. The horizontal and vertical diameters of the FAZ were also calculated. All FAZ measurements were independently performed by two experienced ophthalmologists who were masked to the clinical data, and the measurements showed excellent agreement. The vascular density of the area within 300 microns around the center of the FAZ was defined as the foveal density. Superficial retinal capillary plexus vessel density was determined using the device’s software.

To assess the superficial capillary retinal plexus, the area from the internal limiting membrane (ILM) to 2.6 μm below and up to 15.6 μm below the inner plexiform layer (IPL) was scanned. For deep retinal capillary plexus evaluation, the area from 15 μm below the IPL to 70.2 μm below the IPL was manually scanned.

Optic disk vessel density measurement was automatically performed by the device in the area from 2.6 μm below the ILM to 15.6 μm below the IPL.

The normality of the data distribution was assessed using the graphical methods (histograms, Q‐Q plots, and Box plots) and the Shapiro–Wilk test. For comparisons of two normally distributed quantitative groups, Student’s *t*‐test was used; paired sample comparisons were performed using the paired sample *t*‐test. For comparisons among three or more groups, one‐way ANOVA was used, followed by the Bonferroni test to identify the group responsible for any significant differences. For non‐normally distributed variables, the Mann–Whitney *U* test was used for two‐group comparisons, while the Kruskal–Wallis test was employed for three or more groups, followed by the Dunn test to determine the group responsible for the differences. Chi‐square and Fisher Freeman–Halton tests were used for comparisons of qualitative data. To account for intrasubject correlation arising from the inclusion of both eyes of the same patient, linear mixed‐effects models were applied with subject ID included as a random effect. To control for multiple comparisons and reduce the risk of Type I error, *p* values were adjusted using the Benjamini–Hochberg false discovery rate correction. The primary outcomes of the study were mean superficial capillary plexus (SCP), deep capillary plexus (DCP), and peripapillary vessel density. All sectoral and quadrant‐based analyses were considered secondary and exploratory. Results were considered statistically significant at the *p* < 0.05 level with a 95% confidence interval.

## 3. Results

The eyes of patients diagnosed with unilateral pseudoexfoliation were classified as follows: The eyes with detected pseudoexfoliation (*n* = 39) were designated as Group 1, and the eyes without pseudoexfoliation (*n* = 38) were designated as Group 2. The eyes of 27 patients diagnosed with bilateral pseudoexfoliation (*n* = 54) were classified as Group 3. The eyes of 25 healthy volunteers in the control group (*n* = 50) were classified as Group 4. There were no statistically significant differences in the gender and age distributions among the groups (*p* > 0.05). However, statistically significant differences were found between the stages of glaucoma in the cases with glaucoma (*p* = 0.015; *p* < 0.05). In Group 1, 69.2% of the cases (*n* = 27) were diagnosed with PXS, while 30.8% (*n* = 12) had pseudoexfoliation glaucoma (PXG). Among the patients diagnosed with glaucoma, disease severity was classified as early stage in 25% (*n* = 3), moderate stage in 25% (*n* = 3), and advanced stage in 50% (*n* = 6). Medical treatment was being used by 23.1% of the patients (*n* = 9) with unilateral pseudoexfoliation. In Group 2, glaucoma was diagnosed in 12 eyes. Evaluation of glaucoma severity revealed that 66.7% (*n* = 8) of these eyes were in the mild stage, 16.7% (*n* = 2) in the moderate stage, and 16.7% (*n* = 2) in the advanced stage. Medical therapy was used in 8 eyes (21.1%). In Group 3, among 54 eyes of 27 patients, 63% (*n* = 34) were diagnosed with PXS and 37% (*n* = 20) with PXG. When glaucoma severity was assessed, 65% (*n* = 13) of the eyes were classified as mild, 30% (*n* = 6) as moderate, and 5% (*n* = 1) as advanced. Medical treatment was administered in 18 eyes (33.3%). Comparison of the descriptive characteristics of the groups is presented in Table 1.

**TABLE 1:** Comparison of the descriptive characteristics.

		Group 1 (*n* = 39)	Group 2 (*n* = 38)	Group 3 (*n* = 57)	Group 4 (*n* = 50)	*p*
Gender	Male	18 (46.2)		13 (48.1)	10 (40.0)	^a^0.826
	Female	21 (53.8)		14 (51.9)	15 (60.0)	

Age	Mean ± SD	71.58 ± 6.33		73.19 ± 6.85	71.72 ± 7.86	^d^0.250
	Median	72.5 (54–85)		74 (59–85)	73 (50–85)	

Glaucoma severity	Mild	3 (25.0)	8 (66.7)	13 (65.0)	—	^**c**^ **0.015** ^**∗**^
	Moderate	3 (25.0)	2 (16.7)	6 (30.0)	—	
	Severe	6 (50.0)	2 (16.7)	1 (5.0)	—	

Drug use	No	30 (76.9)	30 (78.9)	36 (66.7)	50 (100)	^**a**^ **0.001** ^**∗∗**^
	Yes	9 (23.1)	8 (21.1)	18 (33.3)	0 (0)	

*Note:* Bold values indicate statistically significant results.

^a^Pearson Chi‐square test.

^c^Fisher–Freeman–Halton test.

^d^Student‐*t* test.

^∗^ *p* < 0.05 indicates statistical significance.

^∗∗^ *p* < 0.01 indicates statistical significance.

The analysis of the ocular data from the groups is presented in Table 2. IOP values and cup‐to‐disc ratios differed significantly between the groups (*p* = 0.009). Pairwise comparisons performed to identify the source of this difference revealed that IOP values were significantly lower in the control group than in the group with unilateral pseudoexfoliation (*p* = 0.004), and that the cup‐to‐disc ratio was significantly lower in the control group compared with the groups in which pseudoexfoliation was detected (*p* = 0.001).

**TABLE 2:** Comparison of ocular parameters across groups.

		Group 1 (*n* = 39)	Group 2 (*n* = 38)	Group 3 (*n* = 54)	Group 4 (*n* = 50)	*p*
BCVA (logmar)	Mean ± SD	0.14 ± 0.12	0.11 ± 0.11	0.19 ± 0.13	0.14 ± 0.14	^d^0.249
	Median	0.2 (0–0.5)	0.1 (0–0.3)	0.2 (0–0.5)	0.1 (0–0.3)	

IOP (mmHg)	Mean ± SD	14.46 ± 2.33	13.63 ± 3.08	14.28 ± 3.24	13.02 ± 1.45	^**e**^ **0.009** ^**∗∗**^
	Median	14 (9–20)	14 (9–20)	14 (9–24)	13 (11–16)	

Cupping (c/d)	Mean ± SD	0.37 ± 0.19	0.31 ± 0.14	0.38 ± 0.19	0.24 ± 0.07	^**e**^ **0.001** ^**∗∗**^
	Median	0.3 (0.2–0.9)	0.3 (0.2–0.9)	0.3 (0.2–0.9)	0.2 (0.2–0.4)	

CMT (μm)	Mean ± SD	537.46 ± 28.30	538.08 ± 30.30	547.00 ± 23.04	547.52 ± 23.74	^d^0.149
	Median	540 (448–634)	538 (439–644)	545 (500–600)	545 (500–600)	

Axial length (mm)	Mean ± SD	22.29 ± 1.74	23.23 ± 1.40	22.91 ± 0.74	22.94 ± 0.65	^e^0.116
	Median	22.9 (21–25)	22.8 (21–24.6)	22.9 (21.6–24.9)	22.9 (21.4–24.4)	

Spherical equivalent (diopter)	Mean ± SD	−0.80 ± 1.73	−0.25 ± 1.10	−0.45 ± 0.49	−0.36 ± 0.74	^e^0.116
	Median	−0.75 (−3/3)	−0.125 (−2/2.50)	0.31 (−2125/3)	−0.5 (−1.8/1.1)	

*Note:* Bold values indicate statistically significant results. IOP: intraocular pressure.

Abbreviations: BCVA, best corrected visual acuity; CMT, central macular thickness.

^d^One‐way ANOVA test.

^e^Kruskal–Wallis test.

^∗∗^ *p* < 0.01 indicates statistical significance.

Statistically significant differences were observed in the SCP measurements across groups in various quadrants. In the upper perifoveal quadrant, comparisons yielded a significant difference (*p* = 0.027; *p* < 0.05), with the control group exhibiting higher SCP measurements than Group 3 (*p* = 0.020; *p* < 0.05). In the lower perifoveal quadrant, similar findings emerged (*p* = 0.019; *p* < 0.05), where the control group had higher measurements than the unilateral pseudoexfoliation group (Group 1) (*p* = 0.012; *p* < 0.05). The nasal perifoveal quadrant also showed significant differences (*p* = 0.012; *p* < 0.05), with Group 3 measuring significantly higher than the nonpseudoexfoliation group (*p* = 0.046; *p* < 0.05). Moreover, in the temporal perifoveal quadrant, significant differences were noted (*p* = 0.005; *p* < 0.01), where the control group had higher measurements than both Group 1 and Group 2 (*p* = 0.023; *p* = 0.009; *p* < 0.05). Significant differences in the foveal region were recorded (*p* = 0.001; *p* < 0.01), with control group measurements being significantly greater than those in all other groups (*p* = 0.001; *p* = 0.005; *p* = 0.007; *p* < 0.01). Lastly, average SCP measurements across groups were significantly different (*p* = 0.001; *p* < 0.01), with the control group having higher values than the other groups (*p* = 0.001; *p* = 0.001; *p* = 0.005; *p* < 0.01). The vascular density measurements of the SCP are detailed in Table 3.

**TABLE 3:** Vascular density measurements of the macular superficial capillary plexus.

		Group 1 (*n* = 39)	Group 2 (*n* = 38)	Group 3 (*n* = 54)	Group 4 (*n* = 50)	^d^ *p*
VD upper	Mean ± SD	45.71 ± 4.25	45.72 ± 4.15	45.07 ± 4.28	47.26 ± 1.93	**0.027** ^**∗**^
	Median	45.7 (34.6–53.2)	45.6 (34.6–54.2)	45.1 (25.2–54.4)	47.3 (42.7–50.7)	

VD lower	Mean ± SD	43.78 ± 4.34	45.6 ± 3.26	45.11 ± 5.42	46.49 ± 2.20	**0.019** ^**∗**^
	Median	44.5 (35.9–52.6)	45.9 (35.2–52.5)	45.1 (24.9–57.5)	46.7 (41.5–51.2)	

VD nasal	Mean ± SD	43.48 ± 4.32	43.35 ± 3.64	45.48 ± 4.24	44.76 ± 1.52	**0.012** ^**∗**^
	Median	43.1 (32.1–53.6)	42.6 (37.5–51.3)	45.3 (35.5–56.7)	44.6 (41–49.9)	

VD temporal	Mean ± SD	43.73 ± 5.12	43.49 ± 3.48	44.85 ± 4.13	46.11 ± 1.99	**0.005** ^**∗∗**^
	Median	43.4 (31.3–56.3)	44.1 (34.5–49.4)	44.8 (34.7–57.5)	46.1 (42.3–51.3)	

VD foveal	Mean ± SD	20.38 ± 6.38	21.8 ± 5.26	22.33 ± 6.81	26.04 ± 4.18	**0.001** ^**∗∗**^
	Median	18.8 (14.8–31.9)	19.8 (13.8–36)	21.9 (10.4–39.3)	25.8 (17.2–34.3)	

Average measurements	Mean ± SD	39.42 ± 3.18	39.99 ± 2.36	40.57 ± 2.35	42.13 ± 1.21	**0.001** ^**∗∗**^
	Median	39.5 (34.8–53.1)	39.7 (36.1–45.4)	40.2 (35.3–47.4)	42.2 (39.7–45.7)	

*Note:* Bold values indicate statistically significant results.

Abbreviation: VD, vascular density.

^d^One‐way ANOVA test and Bonferroni test.

^∗^ *p* < 0.05 indicates statistical significance.

^∗∗^ *p* < 0.01 indicates statistical significance.

Significant differences in DCP vascular density were observed across groups in the upper perifoveal quadrant (*p* = 0.015). Post hoc analysis showed that values in the unilateral pseudoexfoliation group were significantly lower than those in Group 3 and controls (*p* = 0.023; *p* = 0.041). In the lower perifoveal quadrant, DCP vascular density also differed significantly (*p* = 0.016). Pairwise comparisons indicated higher values in the nonpseudoexfoliation group compared to pseudoexfoliation groups (*p* = 0.019). No significant differences were detected in the nasal perifoveal quadrant (*p* > 0.05). However, in the temporal perifoveal quadrant, significant differences were found (*p* = 0.037), with control group measurements exceeding those of Group 2 (*p* = 0.044). In the foveal region, vascular density differed significantly (*p* = 0.016), with higher values in controls compared to Group 1 (*p* = 0.012). DCP vascular density metrics for all groups are summarized in Table 4.

**TABLE 4:** Vascular density measurements of the macular deep capillary plexus.

		Group 1 (*n* = 39)	Group 2 (*n* = 38)	Group 3 (*n* = 54)	Group 4 (*n* = 50)	^d^ *p*
VD upper	Mean ± SD	46.62 ± 5.31	49.28 ± 4.71	49.55 ± 5.83	49.41 ± 2.59	**0.015** ^**∗**^
	Median	47.6 (33.7–58.5)	50.4 (36.1–56.1)	50.4 (29.4–64.9)	49.5 (44.6–55)	

VD lower	Mean ± SD	46.41 ± 5.86	49.92 ± 5.12	47.84 ± 6.02	49.08 ± 3.1	**0.016** ^**∗**^
	Median	47.5 (31.2–54.6)	50.6 (37.6–60.9)	48.9 (24.3–58.2)	49 (44.2–56)	

VD nasal	Mean ± SD	46.68 ± 3.56	47.38 ± 4.31	48.57 ± 4.32	46.76 ± 2.54	**0.056**
	Median	46.5 (40.8–56)	47 (38.4–55.8)	48.2 (39.8–58.2)	46.7 (40.6–55.4)	

VD temporal	Mean ± SD	47.11 ± 3.82	45.66 ± 3.23	47.52 ± 4.51	47.71 ± 1.85	**0.037** ^**∗**^
	Median	47.5 (38.2–57.6)	45.1 (40.3–53)	46.8 (36.7–65.4)	47.8 (44.1–51.5)	

VD foveal	Mean ± SD	22.72 ± 7.22	26.23 ± 8.77	24.98 ± 7.47	27.36 ± 3.87	**0.016** ^**∗**^
	Median	21.2 (10.2–47.7)	24.4 (14.9–56.3)	24.5 (11.5–43.5)	27.6 (20.3–33.8)	

Average measurements	Mean ± SD	41.91 ± 2.84	43.69 ± 2.71	43.69 ± 2.70	44.06 ± 1.67	**0.001** ^**∗∗**^
	Median	41.8 (35.7–49.4)	43.3 (36.7–50.9)	43.5 (36.9–50.1)	44.2 (40.6–48.2)	

*Note:* Bold values indicate statistically significant results.

Abbreviation: VD, vascular density.

^d^One‐way ANOVA test and Bonferroni test.

^∗^ *p* < 0.05 indicates statistical significance.

^∗∗^ *p* < 0.01 indicates statistical significance.

Significant differences were observed in the FAZ area among the groups (*p* = 0.001). Post hoc analysis revealed that FAZ area measurements in the control group were significantly smaller than those in the other three groups (*p* = 0.001; *p* = 0.027). Horizontal FAZ diameter also differed significantly among groups (*p* = 0.005), with smaller values in the control group compared to Groups 1, 2, and 3 (*p* = 0.005). Similarly, vertical FAZ diameter measurements differed significantly (*p* = 0.001). The control group showed significantly smaller vertical diameters compared to the other groups (*p* = 0.001; *p* = 0.036; *p* = 0.001). FAZ parameters for all groups are detailed in Table 5.

**TABLE 5:** Foveal avascular zone parameters.

		Group 1 (*n* = 39)	Group 2 (*n* = 38)	Group 3 (*n* = 54)	Group 4 (*n* = 50)	^d^ *p*
FAZ area (mm^2^)	Mean ± SD	0.28 ± 0.08	0.25 ± 0.08	0.26 ± 0.08	0.21 ± 0.08	**0.001** ^**∗∗**^
	Median	0.3 (0.2–0.5)	0.2 (0.1–0.4)	0.3 (0.1–0.5)	0.2 (0.1–0.4)	

Horizontal FAZ diameter (mm)	Mean ± SD	0.56 ± 0.14	0.54 ± 0.1	0.54 ± 0.14	0.48 ± 0.10	**0.005** ^**∗∗**^
	Median	0.6 (0.2–0.9)	0.5 (0.3–0.7)	0.5 (0.3–0.8)	0.5 (0.3–0.7)	

Vertical FAZ diameter (mm)	Mean ± SD	0.57 ± 0.12	0.52 ± 0.12	0.54 ± 0.15	0.44 ± 0.11	**0.001** ^**∗∗**^
	Median	0.6 (0.3–0.8)	0.5 (0.2–0.7)	0.5 (0.3–0.8)	0.5 (0.2–0.7)	

*Note:* Bold values indicate statistically significant results.

Abbreviation: FAZ, foveal avascular zone.

^d^One‐way ANOVA test and Bonferroni test.

^∗∗^ *p* < 0.01 indicates statistical significance.

Significant differences were observed in upper peripapillary quadrant vessel density among the groups (*p* = 0.001). Post hoc analysis showed that control group values were significantly higher than those in the pseudoexfoliation groups (*p* = 0.021; *p* = 0.001). Lower peripapillary quadrant vessel density also differed significantly (*p* = 0.001), with measurements in the unilateral pseudoexfoliation group significantly lower than those in Group 3 and the control group (*p* = 0.024; *p* = 0.001). No significant differences were noted in nasal peripapillary quadrant vessel density (*p* > 0.05). However, temporal peripapillary quadrant vessel density differed significantly (*p* = 0.001), with control group values higher than those in the unilateral pseudoexfoliation group (*p* = 0.001). Intrapapillary vessel density measurements showed significant differences (*p* = 0.001), with control group values exceeding those of Groups 1, 2, and 3 (*p* = 0.001; *p* = 0.004). Peripapillary vascular density results are summarized in Table 6.

**TABLE 6:** Peripapillary vascular density measurements.

		Group 1 (*n* = 39)	Group 2 (*n* = 38)	Group 3 (*n* = 54)	Group 4 (*n* = 50)	^d^ *p*
VD upper	Mean ± SD	51.80 ± 6.90	52.59 ± 5.70	49.91 ± 7.30	55.93 ± 5.98	**0.001** ^**∗∗**^
	Median	52.2 (36.1–66.3)	53.3 (40.3–64.3)	50.4 (34.2–64.9)	56.6 (39–68.5)	

VD lower	Mean ± SD	50.21 ± 7.93	53.07 ± 5.63	54.04 ± 6.08	56.23 ± 5.31	**0.001** ^**∗∗**^
	Median	53.8 (34.3–61)	52.1 (40.3–68)	54.1 (36.8–66.2)	56.3 (38.3–65.3)	

VD nasal	Mean ± SD	46.21 ± 5.66	46.85 ± 5.87	47.43 ± 6.84	49.12 ± 7.28	0.178
	Median	46 (34.3–59.1)	46.5 (37.8–56.7)	47.3 (31.6–59.8)	48.8 (30.4–69.3)	

VD temporal	Mean ± SD	44.98 ± 6.51	46.89 ± 5.10	47.34 ± 5.23	50.15 ± 6.46	**0.001** ^**∗∗**^
	Median	46.6 (31.1–57.5)	47.1 (36.7–57.2)	47.5 (35.7–59.9)	50.7 (33.5–63.4)	

İntrapapillary VD	Mean ± SD	35.16 ± 11.70	40.46 ± 9.04	38.36 ± 10.39	44.88 ± 6.72	**0.001** ^**∗∗**^
	Median	36.4 (14–55.4)	39.8 (21–53)	39.2 (19–63.5)	45.6 (26.9–57.8)	

*Note:* Bold values indicate statistically significant results.

Abbreviation: VD, vascular density.

^d^One‐way ANOVA test and Bonferroni test.

^∗∗^ *p* < 0.01 indicates statistical significance.

The correlation between FAZ area and macular SCP and DCP vessel density measurements is shown in Table 7. No statistically significant relationship was found between the FAZ area and the average macular SCP vessel density (*p* > 0.05). However, a weak but statistically significant negative correlation was observed between the FAZ area and the average macular DCP vessel density (i.e., as DCP vessel density decreased, FAZ area increased) (*r* = −0.234; *p* = 0.007).

**TABLE 7:** The relationship between FAZ area and macular density.

	**FAZ area**	
	***r***	**p**

SCP VD	−0.113	0.131
DCP VD	−0.234	**0.007^∗∗^**

*Note:* Bold values indicate statistically significant results. r = Pearson’s correlation test.

Abbreviations: DCP, deep capillary plexus; SCP, superficial capillary plexus; VD, vascular density.

^∗∗^ *p* < 0.01.

Subgroup analysis comparing eyes with PEX and PXG revealed no statistically significant differences in functional or vascular parameters. Visual field indices, including MD (*p* = 0.485), pattern standard deviation (PSD) (*p* = 0.910), and glaucoma stage distribution (*p* = 0.273), were comparable between the two groups. Furthermore, no significant differences were observed between PEX and PXG eyes in terms of average SCP vessel density (*p* = 0.163), average DCP vessel density (*p* = 0.055), FAZ area (*p* = 0.733), horizontal FAZ diameter (*p* = 0.765), vertical FAZ diameter (*p* = 0.627), or intrapapillary vessel density (*p* = 0.933). A detailed comparison of functional and vascular parameters between PEX and PXG eyes is presented in Table 8.

**TABLE 8:** Comparison of functional and OCT angiography parameters between eyes with pseudoexfoliation syndrome and pseudoexfoliative glaucoma.

		PEX (*n* = 61)	PXG (*n* = 32)	*p*
Visual field test MD	Mean ± SD	−6.49 ± 8.4	−9.28 ± 8.1	^b^0.485
	Median	−3.1 (−21.2‐‐0.3)	−6.5 (−26.7‐‐0.5)	

Visual field test PSD	Mean ± SD	5.3 ± 3.45	5.09 ± 3.79	^b^0.910
	Median	4.7 (1.5–10.6)	3.4 (1.5–13.7)	

Glaucoma severity	Mild	4 (80.0)	12 (42.9)	^c^0.273
	Moderate	0 (0)	9 (32.1)	
	Severe	1 (20.0)	7 (25.0)	

SCP average VD	Mean ± SD	40.38 ± 2.88	39.53 ± 2.51	0.163
	Median	40.1 (34.8–53.1)	39 (35.2–44.4)	

DCP average VD	Mean ± SD	43.36 ± 2.78	42.15 ± 2.96	0.055
	Median	43.2 (35.7–50.1)	42.7 (36.1–48.2)	

FAZ area (mm^2^)	Mean ± SD	0.26 ± 0.07	0.27 ± 0.09	0.733
	Median	0.3 (0.1–0.5)	0.3 (0.1–0.5)	

Horizontal FAZ diameter (mm)	Mean ± SD	0.55 ± 0.12	0.56 ± 0.16	0.765
	Median	0.6 (0.2–0.8)	0.5 (0.3–0.9)	

Vertikal FAZ diameter (mm)	Mean ± SD	0.55 ± 0.13	0.56 ± 0.15	0.627
	Median	0.5 (0.3–0.8)	0.6 (0.3–0.8)	

İntrapapillary VD	Mean ± SD	36.95 ± 10.6	37.16 ± 11.92	0.933
	Median	36.9 (16.8–63.5)	37 (14–62.7)	

Abbreviations: DCP, deep capillary plexus; SCP, superficial capillary plexus; VD, vascular density.

^b^Mann–Whitney *U* test.

^c^Fisher–Freema–Halton test.

These findings indicate that microvascular alterations are present in eyes with pseudoexfoliation; however, the potential contribution of concomitant glaucoma cannot be excluded.

## 4. Discussion

In this study, we utilized OCT‐A to investigate macular and peripapillary microvascular alterations in patients with unilateral and bilateral pseudoexfoliation. Our primary outcome measures—mean macular SCP and DCP densities, and mean peripapillary vessel density—demonstrated significant reductions in all PXS groups compared to healthy controls. Microvascular alterations were also detected in the fellow eyes of patients with clinically unilateral PXS. These findings suggest that microvascular involvement may extend beyond clinically apparent disease; however, the presence of concomitant glaucoma should be taken into account when interpreting bilateral involvement.

To ensure the validity of our results, we matched our cohorts for age and gender, as macular density is known to decline progressively after the fifth decade [6, 7] although it remains largely independent of gender in most normative databases [8, 9]. Furthermore, we controlled for refractive errors and axial length to avoid bias in OCT‐A measurements, as high myopia has been associated with expanded FAZ areas and reduced vascular density [10, 11].

The confounding effect of glaucoma represents an important consideration in the interpretation of the present findings, as IOP, glaucomatous structural damage, and antiglaucomatous treatment have been shown to influence OCT‐A–derived vascular parameters [12, 13]. Although IOP was comparable between the groups and a subgroup analysis between PEX and PXG eyes did not reveal statistically significant differences in the measured parameters, glaucoma status and severity were not incorporated as covariates in the primary statistical models. Therefore, the observed microvascular alterations may reflect the combined effects of PXS and concomitant glaucoma rather than PXS alone. In the unilateral pseudoexfoliation group, fellow eyes were classified according to the presence of glaucoma rather than the clinical detection of pseudoexfoliative material. Consequently, these eyes should not be considered truly unaffected control eyes, and this classification may have influenced the interpretation of bilateral microvascular involvement.

In our study, peripapillary and intrapapillary vessel density was found to be significantly lower in both eyes of patients with unilateral pseudoexfoliation and in patients with bilateral pseudoexfoliation compared to the control group, except for the nasal quadrant (*p* < 0.05). This sectorial pattern is consistent with the findings of Safizadeh et al., who reported that the most significant decreases in optic nerve head density occurred in the temporal and inferior sectors in PXS [14]. The preservation of the nasal sector may be related to regional anatomical differences or varying susceptibility to the accumulation of the pseudoexfoliative material. The microvascular loss observed in the contralateral, clinically unaffected eyes is further supported by experimental models by Rojas et al., which demonstrated bilateral glial and inflammatory activation even in unilateral ocular stress [15].

Macular analysis revealed widespread reduction in both SCP and DCP densities. This aligns with previous studies by Çınar et al. [16] and Güngör et al. [17] who reported significantly lower parafoveal and foveal densities in PXS‐positive eyes. Furthermore, we identified a significant negative correlation (*r* = −0.234) between FAZ area and DCP density. As the DCP is solely reliant on capillary perfusion, it may be more vulnerable to the chronic ischemic stress or structural vasculopathy associated with pseudoexfoliation than the more robustly supplied SCP [18].

The presence of microvascular changes in the fellow eyes of unilateral PXS patients is a critical finding. In our cohort, a portion of these eyes was diagnosed with PXG despite the lack of visible exfoliative material. This observation may indicate that microvascular alterations can be detected in clinically non‐PEX eyes; however, the influence of coexisting glaucoma should be considered when interpreting these findings.

The main strength of this study is its comprehensive quadrant‐based analysis and the use of linear mixed‐effects models to account for intereye correlation. However, the cross‐sectional design of this study means that causal or temporal relationships between pseudoexfoliation, microvascular alterations, and glaucomatous progression cannot be definitively established.

In conclusion, PXS is associated with widespread macular and peripapillary microvascular compromise; however, the potential confounding effect of concomitant glaucoma should be taken into account. These findings highlight the clinical value of OCT‐A as a complementary tool for the early detection and monitoring of subclinical bilateral involvement in PXS.

## 5. Conclusion

In conclusion, our study demonstrates that PXS is associated with widespread macular and peripapillary microvascular alterations. These changes were observed in both eyes of patients with clinically unilateral disease as well as in patients with bilateral pseudoexfoliation. However, because glaucoma status and severity were not incorporated as covariates in the primary statistical models, the contribution of concomitant glaucoma to the observed OCT‐A findings cannot be fully excluded. OCT‐A may represent a useful adjunctive tool for the evaluation of microvascular involvement in PXS, but longitudinal studies with appropriate adjustment for glaucoma‐related factors are required to clarify the independent vascular effects of pseudoexfoliation.

### 5.1. Limitations

Although glaucoma severity and treatment profiles were descriptively reported, glaucoma status and severity were not incorporated as covariates in the primary statistical models. Therefore, the potential confounding effect of glaucoma on OCT‐A metrics cannot be fully excluded.

## Author Contributions

MD Arzu Karakiraz contributed to the conception, study design, data acquisition, analysis, and drafting of the manuscript. Associate Professor Dr. Şerife Bayraktar provided critical input in study conception, interpretation of findings, and substantial revision of the manuscript for important intellectual content.

## Funding

This research did not receive any specific grant from funding agencies in the public, commercial, or not‐for‐profit sectors.

## Disclosure

Both authors approved the final version of the manuscript and agree to be accountable for all aspects of the work.

## Conflicts of Interest

The authors declare no conflicts of interest.

## Data Availability

The data that support the findings of this study are available from the corresponding author upon reasonable request.
